# Adaptation of community health worker-delivered behavioral activation for torture survivors in Kurdistan, Iraq

**DOI:** 10.1017/gmh.2015.22

**Published:** 2015-12-21

**Authors:** J. F. Magidson, C. W. Lejuez, T. Kamal, E. J. Blevins, L. K. Murray, J. K. Bass, P. Bolton, S. Pagoto

**Affiliations:** 1 Department of Psychiatry, Massachusetts General Hospital (MGH)/Harvard Medical School, Boston, MA, USA; 2 Department of Psychology, Center for Addictions, Personality, and Emotion Research (CAPER), University of Maryland, College Park, MD, USA; 3 Fine Arts Institute, University of Sulaimani, Kurdistan Region, Iraq; 4 Department of Mental Health, Johns Hopkins Bloomberg School of Public Health, Baltimore, MD, USA; 5 Center for Refugee and Disaster Response and Department of International Health, Johns Hopkins Bloomberg School of Public Health, Baltimore, MD, USA; 6 Division of Preventive and Behavioral Medicine, Department of Medicine, University of Massachusetts Medical School, Worcester, MA, USA

**Keywords:** Adaptation, behavioral activation, depression, task shifting, trauma

## Abstract

**Background.:**

Growing evidence supports the use of Western therapies for the treatment of depression, trauma, and stress delivered by community health workers (CHWs) in conflict-affected, resource-limited countries. A recent randomized controlled trial (Bolton *et al*. 2014*a*) supported the efficacy of two CHW-delivered interventions, cognitive processing therapy (CPT) and brief behavioral activation treatment for depression (BATD), for reducing depressive symptoms and functional impairment among torture survivors in the Kurdish region of Iraq.

**Methods.:**

This study describes the adaptation of the CHW-delivered BATD approach delivered in this trial (Bolton *et al*. 2014*a*), informed by the Assessment–Decision–Administration-Production–Topical experts–Integration–Training–Testing (ADAPT–ITT) framework for intervention adaptation (Wingood & DiClemente, 2008). Cultural modifications, adaptations for low-literacy, and tailored training and supervision for non-specialist CHWs are presented, along with two clinical case examples to illustrate delivery of the adapted intervention in this setting.

**Results.:**

Eleven CHWs, a study psychiatrist, and the CHW clinical supervisor were trained in BATD. The adaptation process followed the ADAPT–ITT framework and was iterative with significant input from the on-site supervisor and CHWs. Modifications were made to fit Kurdish culture, including culturally relevant analogies, use of stickers for behavior monitoring, cultural modifications to behavioral contracts, and including telephone-delivered sessions to enhance feasibility.

**Conclusions.:**

BATD was delivered by CHWs in a resource-poor, conflict-affected area in Kurdistan, Iraq, with some important modifications, including low-literacy adaptations, increased cultural relevancy of clinical materials, and tailored training and supervision for CHWs. Barriers to implementation, lessons learned, and recommendations for future efforts to adapt behavioral therapies for resource-limited, conflict-affected areas are discussed.

## Introduction

In resource-limited countries, a substantial gap exists between mental health needs and access to the evidence-based care to address those needs (Patel *et al.* 2010). Lack of mental health infrastructure combined with lack of clinical research results in limited knowledge of how to treat mental health conditions. Evidence-based treatments (EBTs) developed in Western nations can be adapted for resource-limited settings, but such efforts must consider a range of factors including culture, language, resources, infrastructure, and setting (Collins *et al.* 2006; Angotti, 2010).

Kurdistan, Iraq is a low-resource nation with a population that has been subjected to systematic torture and violence, including bombing, chemical warfare, and forced dislocations from 1986 to 1989 (Khateri *et al.*, 2003; Hiltermann, 2007). Individuals who survived these attacks in Kurdistan continue to suffer from poverty, discrimination, stigma, depression, traumatic stress, and reduced social, physical, and economic functioning (Bolton *et al.* 2013). Current unrest in Kurdistan puts many at risk for retraumatization, and limited mental health infrastructure and lack of access to EBTs are of increasing concern.

The evidence base is growing for Western therapies targeting depression, trauma, and stress in resource-limited countries that may be useful in this setting (Bolton *et al.* 2003, 2007, 2014*b*; Rahman *et al.* 2008; Patel *et al.* 2010; Bass *et al.* 2013). For example, Bolton *et al.* (2014*a*) conducted a randomized clinical trial (RCT) examining the efficacy of two Western therapies for reducing depressive symptoms, functional impairment, and symptoms of generalized anxiety, posttraumatic stress, and traumatic grief symptoms among torture survivors in the Kurdish region of Iraq. These therapies included cognitive processing therapy (CPT; Resick & Schnicke, 1992; Kaysen *et al.* 2013) and brief behavioral activation treatment for depression (BATD; Lejuez *et al.* 2011), both delivered by community health workers (CHWs). Results of the trial indicated the efficacy of both approaches (see Bolton *et al.* 2014*a*). Regarding BATD, results supported its efficacy in this context to reduce depressive symptoms and improve functioning, with medium to large effect size estimates for both in comparison to wait-list controls (Bolton *et al.* 2014*a*).

Within any RCTs in low-resource settings, researchers have to examine the processes of adaptation to understand how EBTs can be applied to non-Western populations (Patel *et al.* 2007; Verdeli *et al.* 2008; Kaysen *et al.* 2013; Murray *et al.* 2013; Chowdhary *et al.* 2014). Here we address the process of adaptation of BATD by CHWs for torture survivors in Kurdistan, Iraq from the larger trial (Bolton *et al.* 2014*a*). Adaptation was informed by the Assessment–Decision–Administration–Production–Topical experts–Integration–Training–Testing (ADAPT–ITT) model of intervention adaptation (Wingood & DiClemente, 2008). This model outlines steps for adapting an EBT for delivery in another setting and has been used in international implementation research (Saleh-Onoya *et al.* 2009). The framework begins with recommendations for a formative assessment to guide implementation, and then discusses factors related to the decision of selecting an intervention, and components of the original intervention that may need to be adapted for administration in this context. Next, recommendations for production of the adapted intervention are provided in consultation with topical experts. Moving closer to implementation, a plan is then developed for integration of feedback and training of providers, likely using a task shifting model of care delivery in a resource-limited international care setting, which requires additional adaptation to the intervention and training procedures. Finally, the last phase of this model is testing the adapted intervention in an implementation trial (Wingood & DiClemente, 2008). Consistent with the ADAPT–ITT model, the process of adaptation was iterative. In this paper, we describe cultural modifications to BATD, adaptations for the low-literacy patient population, and tailored training for non-specialist CHWs with little to no prior experience with behavioral theories and therapies. We present two clinical case examples to illustrate the application of the adapted BATD intervention in this setting. Finally, we discuss barriers to adaptation and training, lessons learned, and provide suggestions for similar efforts to adapt and implement behavioral therapies in resource-limited settings.

## Method

### Formative assessment and intervention selection

A qualitative, formative assessment with survivors of torture in Kurdistan was first conducted to understand local perceptions of mental health needs and to guide intervention selection and adaptation for a subsequent RCT. Initial qualitative results with 63 survivors demonstrated that respondents were reporting symptoms consistent with acute stress disorders, posttraumatic stress disorder (PTSD) and depression, as well as psychosocial distress related to familial relationships and societal belongingness (see Bolton *et al.* 2013 for greater detail).

Intervention selection was guided by the formative assessment. First, the formative assessment suggested that although trauma symptoms were reported, depression and psychosocial distress were of greater concern in this population (Bolton *et al.* 2013). Second, it was important to identify an evidence-based behavioral treatment for depression that could be delivered in a brief format. Third, it was necessary to select an intervention that could be feasible for delivery by paraprofessional providers. Fourth, it was essential to select an intervention that did not seem to conflict with Kurdish culture or practices and did not contract local perceptions of the causes of depression, and one that was amenable to cultural adaptations and modifications for those with limited education and literacy issues.

### Behavioral activation treatment for depression

Behavioral activation is an evidence-based behavioral therapy that was originally developed for the treatment of depression in the USA (Martell *et al.* 2001; Dimidjian *et al.* 2006; Cuijpers *et al.* 2007; Lejuez *et al.* 2011). Brief BATD is a straightforward version of behavioral activation that uses a present-focused, stepwise treatment approach based on reinforcement theory (Ferster, 1973; Lewinsohn, 1974) to help individuals develop a more fulfilling, values-driven life (for a description and differentiation from other BA therapies, see Hopko *et al.* 2003; Lejuez *et al.* 2011). BATD is a client-centered, tailored approach in which the therapist helps the client identify and schedule rewarding activities related to the client's self-identified personal values. Psychoeducation, self-monitoring of daily activities, and behavioral contracting are additional components of the treatment (see Lejuez *et al*. 2011 for greater detail).

Behavioral activation approaches are considered efficacious for the treatment of depression in Western countries (Cuijpers *et al.* 2007; Ekers *et al.* 2008; Mazzucchelli *et al.* 2009; Sturmey, 2009). In recent years, the application of BATD has expanded to other conditions including anxiety (Hopko *et al.* 2011), other stress-related disorders (Acierno *et al.* 2012; Strachan *et al.* 2012), substance use (MacPherson *et al.* 2010; Magidson *et al.* 2011; Reynolds *et al.* 2011), and HIV medication adherence (Daughters *et al.* 2010; Magidson *et al.* 2014). Because the treatment is guided by the client's unique values, it lends itself to implementation for diverse cultural contexts (Collado *et al.* 2013) and has previously been adapted for individuals with limited education and literacy issues (Daughters *et al*. 2008; Magidson *et al.* 2011). Evidence exists for implementation in a wide range of clinical settings and provider types, including both specialized and non-specialized mental health staff, such as mental health nurses and primary care workers with no professional training in behavioral therapy (Ekers *et al.* 2011*b*, 2013; Moradveisi *et al.* 2013). In resource-limited global settings, accumulating evidence supports the delivery of BATD by CHWs, such as lay HIV adherence counselors and nurses in sub-Saharan Africa (Chibanda *et al.* 2011; Andersen *et al.* 2012). Even with a strong theoretical basis, the delivery of BATD does not require the provider to have training in complex principles of behavior analysis, personality, unconscious motives, or cognition. Additionally, although it is not a trauma-focused intervention, pilot studies using BATD with trauma-affected populations in the USA have demonstrated improvements in PTSD symptoms (Mulick & Naugle, 2004; Jakupcak *et al.* 2006; Jakupcak *et al.* 2010; Wagner *et al.* 2007; Turner & Jakupcak, 2010).

### Treatment adaptation

Treatment adaptation occurred over the course of the study using a highly inclusive approach, such that study staff, supervisors, CHWs, and patients (via CHWs) played an important role throughout all phases of adaptation. Moreover, the process was iterative where feedback was sought after changes were made and additional changes followed. This process repeated until consensus was reached.

### Adaptations prior to training

Based on the qualitative work by Bolton *et al*. (2013) conducted in the region, select adaptations were made prior to arriving in Kurdistan. These adaptations included tailoring BATD for low literacy, cultural modifications, including modifying the symptom picture of depression to include symptoms specific to Kurdish culture, and designing training for non-specialist CHW providers, which included translating and interpreting behavioral concepts. We attempted to make appropriate cultural references in the training material. For instance, where the protocol referred to Western activities, changes were made to reflect common activities in Kurdistan we learned from our partnering organization. Also, modifications were made to accommodate patients with low or no literacy and writing skills. The language of the original manual was simplified and psychological jargon was removed, and rather than using text-based self-monitoring forms, we developed an image-based format where patients could use stickers with images to track their activities. Although some a priori modifications were made to the training material, we attempted to preserve as much as possible to limit assumptions of what would and would not work in this setting. Additional modifications to the treatment were identified in the training and treatment phases (described below).

### Training procedures

Eleven CHWs, a study psychiatrist, and the CHW clinical supervisor (TK) were brought to a central location training site in Sulaymaniyah for a 7-day training in the concepts, principles, and procedures of BATD. Training was led by two US-based experts in BATD (SP, CL) and was conducted in English with simultaneous Kurdish translation through a professional translator. The local study psychiatrist and the CHW supervisor were bilingual and helped to translate psychological concepts that the translator did not understand. Training covered the conceptual underpinnings of BATD and reviewed the protocol session by session. The training involved didactic sessions, role plays of each strategy, and opportunities for supervised practice. A priori, modifications were made to traditional BATD trainings that the trainers (CL, SP) typically conducted in the USA, including minimizing psychological jargon, less reliance on slides, a greater focus on training general counseling and CBT skills, and an emphasis on role plays and training of supervisors in effective feedback delivery. Training was designed to be tailored to the CHWs’ prior training and experience with behavioral therapies. For 2 years prior to the BATD training, all CHWs received mental health training by Heartland Alliance International. It was a comprehensive mental health curriculum for paraprofessional-level, community mental health workers in Iraqi Kurdistan and Southern Iraq. The curriculum included basic skills of a helping professional such as confidentiality, active listening, empathy, assessment, treatment planning, and psychoeducation, as well as a discussion of traumatic stress, suicide risk, and planning and advocacy.

### Adaptations during training

Throughout the 1-week training, multiple modifications were made to the treatment based on supervisor and CHW feedback. During training, additional concepts the CHWs were not familiar with were identified that were important to review (e.g. the basic structure of behavioral interventions and behavioral aspects of depression), and other culturally irrelevant examples were identified. For the latter, we queried CHWs for culturally relevant suggestions and incorporated those into the treatment.  During training, possible changes were discussed with the group and modified materials based on the discussion were presented during the next training day.

### Intervention delivery and supervision

Following the initial BATD training, 11 CHWs delivered a 12-session course of individually-delivered BATD to torture survivors with elevated depressive symptoms and functional impairment. A total of 147 individuals were randomly assigned to receive BATD in the larger trial (see Bolton *et al.* 2014*a*). Of those that initiated treatment (*n* = 140), 52% completed all 12 sessions, and 28% dropped out before completing nine sessions (Bolton *et al.* 2014*a*). The CHWs were supervised by a bachelors-level bilingual therapist (TK) who had extensive experience with the target population. The supervisor provided weekly individual supervision in person or by phone to each CHW that involved reviewing progress on each case starting with the most challenging cases. When logistical concerns forced supervision to be canceled for that given week, the supervisor addressed issues from both weeks in the subsequent supervision session. Throughout the course of treatment, US-based experts in BATD (SP, CL) had weekly supervision meetings via Skype with the clinical supervisor (TK). They discussed challenges the CHWs were reporting with the protocol, difficult patient situations, and challenges the supervisor was having during supervision, as well as guided ongoing adaptation of the intervention based on CHW and patient feedback (via the CHWs).

### Adaptations during treatment delivery

During treatment, protocol modifications were made in response to challenges and feedback by supervisors and CHWs, and these changes were discussed directly with the supervisor. The supervisor then met (in a group or individually as need) with the therapists to discuss changes or provide additional training where larger scale changes were made. Throughout the process of adaptation, the goal was a balance between cultural relevance, feasibility, and treatment fidelity.

Four primary adaptations were made during the treatment phase. First, we discovered that patients had difficulty understanding the concept of values and the CHWs struggled with clarifying this concept. The values module in the BATD protocol focuses on individual values, but the idea of a value held by an individual as opposed to collective values was often difficult for participants to conceptualize. To clarify this concept, we changed the wording to be about ‘something important to you and your family.’ Tying this idea directly to activities was more understandable to patients.

The second change was the development of a low literacy version of the Activity Monitoring Form, the document that patients use to record their daily activities. To modify this task for low literacy patients, we developed a form that uses 13 stickers to signify common activities including sleeping, eating, working, housecleaning, talking on the phone, watching TV, reading, going to worship, etc. (also see Lejuez *et al.* 2011). This not only accommodated patients with low literacy, but stickers were also perceived as less threatening to family members compared to patients keeping a journal of their activities. Stickers presented a straightforward, visual approach to work around these two barriers to treatment.

Third, we adapted the approach for using ‘contracts’; in the original BATD manual (Lejuez *et al.* 2001), contracts were designed to be signed by the support person the individual identified in treatment. However, in the current setting, the counselors expressed that this would present difficulties for patients where this request could be viewed as inappropriate or not conveying sufficient respect and deference (e.g. asking a family elder). For this reason, we modified the contracts to not require a formal agreement from a support person, and instead to merely serve as a way for patients to consider who in their life might be a support person, and specific ways in which this person could provide support.

Fourth, the 12-session protocol was not feasible for in person delivery for a minority of participants who lived in rural regions. Telephone-delivered therapy was used as an alternative, or the number of sessions was reduced. CHWs were encouraged by supervisors to be flexible with patients for scheduling purposes and modification of the treatment schedule, yet at the same time ensuring that the fundamental components of BATD were still able to be delivered. Although no comparison of in-person vs. telephone delivery was performed, both the supervisor and therapists largely reported telephone sessions were feasible. Table 1 presents the original BATD treatment components and specific adaptations made for this setting.

**Table 1.** Summary of BATD components and modifications made for the local context

Standard BATD components	Need for adaptation	Specific modification
Therapist training	• Limited resources	• Less reliance on slides
	• Providers had limited therapy experience	• Focus on general counseling and CBT training, more role plays and instruction in feedback delivery
Manual	• Culturally irrelevant references	• Included symptoms from Kurdish culture in description of depression
		• Changed Western activities/food to Kurdish pastimes and meals
		• Replaced culturally irrelevant examples with suggestions from providers
Psychoeducation	• CHWs unfamiliar with psychological terms	• Psychological terms explained or eliminated (e.g. negative and positive reinforcement further explained)
Daily activity monitoring	• Low literacy	• Stickers to denote activities rather than text
Life areas, values, and activities	• Individual values unfamiliar in collectivistic culture	• Changed definition of value to ‘something important to you and your family’
Contracts	• Requesting someone sign a contract deemed not appropriate in all cases	• Signing and ‘binding’ aspect of contracts removed
In person therapy	• Limited transportation available in rural area	• Conducted sessions over the phone
		• Shortened number of sessions

### Intervention testing phase

The final ‘testing’ phase of the ADAPT–ITT framework was conducted as a multi-arm randomized trial comparing BATD, CPT, and a supportive counseling program on depression and trauma-related symptoms and functioning among Kurdish torture survivors in Kurdistan. Detailed elsewhere (Bolton *et al*. 2014*a*), the adapted, paraprofessional delivered BATD intervention was effective in reducing depressive symptoms and improving functioning in this population with large effect sizes when compared to a wait-list control (i.e. *d* = 0.84 for depression; *p* < 0.0001). Further, the acceptability of the intervention was reflected by the high rates of patient retention; of the 107 patients who began BATD in the larger trial, 72% completed at least nine sessions (Bolton *et al.* 2014*a*). Psychosocial factors related to dropout of BATD in the larger trial included self-employment, inconsistent work, and the absence of formal education compared with individuals who completed nine or more sessions of treatment (see Bolton *et al.* 2014*a* for greater detail). All procedures, including the process of adapting BATD, were approved by the Johns Hopkins University Institutional Review Board and the University of Sulaimaniyah College of Medicine's Ethical Committee. All participants were provided informed consent prior to participation.

## Lessons learned in the adaptation process

### Lessons learned in training

Prior to the training, the trainers (CL, SP) had conversations with local counselors to understand the patient population. Logistical and financial challenges made it difficult to conduct pre-trial piloting work as it would have required additional visits to Kurdistan and for CHWs to travel to additional meetings. Thus, the team treated the first wave of participants (one patient per CHW) as the pilot wave to identify barriers and make modifications while still moving toward a culturally relevant adaptation of the treatment following an apprenticeship model of training, pilot cases, and supervision (Murray *et al.* 2011). Counselors took ownership of the treatment by being directly involved in the early adaptations. This model of allowing local clinicians and supervisors to take a lead in adapting the EBT also has been recommended elsewhere (Bass *et al.* 2013; Murray *et al.* 2013).

### Lessons learned in supervision by US-based trainers to the local supervisor

Supervision was largely done via Skype in English, which was made difficult by varying quality in the internet connection at the treatment center in Kurdistan. Understanding each other's accents over a poor quality connection hindered effective communication. Supervisors transitioned to text chatting in these instances (e.g. ‘I don't understand, can you write it out?’) which facilitated clearer communication. In instances where there was not a strong enough internet connection for Skype, supervisors used email exchanges to answer questions and discuss issues.

### Lessons learned in supervision by the local supervisor to the local therapists

Regarding the content of supervision, therapists often reported struggling with balancing time spent developing the therapeutic relationship and time spent on protocol. In many cases supervisors, therapists, and patients had expectations that therapy consists of supportive, unstructured conversation. As a result, a protocol-driven, action-oriented therapy was a completely new experience and in some cases required more detailed justification for this approach. For example, some CHWs reported that the structured nature of BATD limited their flexibility in addressing issues patients brought to sessions. In future trainings, we recommend devoting more time to therapist and local supervisor perceptions of and feelings about brief, structured therapeutic approaches and have them practice striking the balance between relationship building and the behavioral strategies in the protocol. Opportunities to practice this balance in training are recommended (Murray *et al.* 2011).

## Clinical case examples to illustrate delivery of the adapted BATD approach

In this next section, we provide two clinical examples of clients treated with the BATD approach to illustrate the delivery of the adapted approach. Some client characteristics have been altered to protect client confidentiality.

### Case 1:

The client, a married woman, presented for BATD exhibiting a wide range of psychological symptoms including sadness, anger, sleep difficulties, fatigue, and anxiety, as well as environmental adversities including neglecting family members and not being able to participate in social gatherings such as condolence ceremonies. The client did not complete high school, and was living with her husband and two children. The client had no previous history of any type of mental health counseling and was exposed to trauma for a long period of time. Specifically, the client participated in the Peshmarga Kurdish rebellion against Saddam's regime, which included observing considerable bloodshed and the loss of several of her own family members. As a consequence of her role in the rebellion, she was displaced to the Iranian border. In the first few sessions of BATD, the client identified values that were important to her and specific activities that mapped onto her values. For example, she reported that intimacy was an important value of hers when it comes to family relationships. She named taking trips and working with family members as activities consistent with this value.  She reported that literacy was a major value when it comes to education. She listed related activities including taking courses on the eradication of illiteracy and starting to read short books.  She established numerous other detailed activity goals in line with her other personal values including helping people, having a compassionate and loving relationship with her husband, learning new information, participating in activities to pass the time in a useful manner, participating in political events, maintaining self-care, attending to her own happiness, achieving calmness, supervising her children, and relating to her siblings in an emphatic manner.  The client was motivated to participate in BATD, but several barriers to treatment existed. Because of her struggles with low literacy, she initially was dependent on her husband to fill out the BATD homework forms. Over time she became comfortable using stickers to record her activities, but even with this option she preferred completing assignments with her husband's assistance. The client reported that back pain prevented her from coming to session, which resulted in biweekly phone sessions in place of in-person sessions. The client also wanted to speak at length about the traumas she had witnessed, which is not a core aspect of the BATD protocol. The CHW spent some time allowing her to speak about the traumas for a portion of the session depending on the other goals for the session (e.g. for 15 min), and then would refocus her on the therapy goals. Despite the challenges, the client responded well to BATD. She completed all her homework assignments, which further promoted a lifestyle congruent with her stated values. The client reported specific positive changes in her life, including caring for her children and improving her social relationships.

### Case 2:

The client, a single woman in her late 30s, presented for BATD exhibiting sadness, anger, poor familial relationships, feeling that her life was ‘boring’, the wish to die, self-hatred, feelings that no one listens to her, and lack of compassion. She also reported recurrent thoughts about harming (and killing) her family members. The client lived with her brother and his family. The client had experienced significant trauma resulting from the Halabja chemical attack, where she lost her parents and was displaced to the town of Hawar with her brother. She was there for 7 months before she went to Iran for chemical treatment. The client visited a psychiatric hospital after the Halabja bombing, where she received psychotropic medication. With the onset of BATD, the client identified her core values and generated specific activities tied to those values.  For instance, she valued compassion, sincerity, loyalty, honesty, and nurturing her brother's children as her own siblings. In accordance, she identified talking on the phone with her family, cooking, and helping them with their work as activities tied to these values. She also specified activities in line with her values of honesty, having good friends, loyalty, sincerity, learning new information, performing jobs appropriately, maintaining good relationships with employers, better self-care, psychological improvement, and completing daily chores. The CHW reported the client was motivated to participate in BATD and completed 12 sessions. Difficulties with her treatment included her brothers’ resistance to her treatment given concerns about stigma including unintended consequences for the family if others became aware of her seeking treatment. This barrier, in addition to her demanding work schedule, hindered her ability to attend weekly sessions. As a solution, her treatment provider who arranged home and work visits spoke with her brother regarding treatment, which resulted in his willingness to allow her to continue treatment. As a result of BATD, the client experienced many positive changes in her life. Specifically, she reported improved relationships with her family, loving herself and the people around her, the desire to live and enjoy life, reduced sadness and anger, and improved job performance. Indeed, her brother witnessed these positive changes, and even asked to be a client himself. The focus of BATD on adherence to her daily goals helped engender the congruence of her lifestyle and value system, her motivation to treat her depression, and her perseverance and commitment to continue treatment despite logistical constraints. Furthermore, her treatment allowed her to obtain a referral to a psychiatrist and be properly diagnosed with epilepsy, which had been misdiagnosed previously.

## Conclusions and next steps

This paper outlined our adaptation of BATD for survivors of torture in Kurdistan, delivered by non-specialist local CHWs, and modifications that were made throughout the training, delivery, and supervision processes. Detailed in the larger trial (Bolton *et al*. 2014*a*), the adapted BATD intervention was found to be acceptable (as evidenced by high rates of treatment retention), and effective in reducing depressive symptoms and improving functioning. Given that the factors most strongly associated with retention were related to employment barriers (Bolton *et al*. 2014*a*), future work should further assess and consider implementation using telemedicine (Egede *et al*. 2009) or mobile phone-based delivery for this approach, particularly where travel and other employment barriers limit therapy attendance.

Lessons learned from adapting and delivering BATD in this setting could inform cultural adaptations of BATD and other brief CBT interventions in resource-limited settings, with varying cultural contexts, resources, and literacy levels. Key areas to consider in implementation of BATD or other related brief behavioral therapies in resource-limited settings include the following: (1) perceptions of self-monitoring by patients and family members; (2) feasibility of image-based self-monitoring to reduce reliance on literacy for treatment and training procedures; (3) consideration of culturally-relevant language, clinical examples, and homework activities to incorporate into the treatment approach, for instance, a primary change was to shift the definition of ‘values’ to be less individualistic, and rather encompass ‘something important to you and your family’; (4) feasibility of and barriers to behavioral contracts, including that perceptions of the ‘signing and binding’ aspects of it may not be appropriate in some cultures or for certain family members, such as elder family members; and (5) appropriateness of the conceptual framework in the culture and the language used to describe it. Finally, if feasible and affordable, pre-pilot testing all procedures would be extremely useful, enabling the trainers to spend more time on the ground with local supervisors and in clinic settings to use this knowledge to inform the approach to training and supervision. Increasing the sample size to allow for a pre-pilot wave that directly precedes the actual study sample may be one way to accomplish this without additional visits for training. Extending the length and depth of training and supervision may be important depending on the expertise and cultural context of local providers.

Finally, Western therapies often involve ‘packages’ of strategies which may make the adaptation process even more complicated. Research that dismantles packages to their active ingredients would be of value to efforts at adaptation in low-resource settings. For example, training CHWs in BATD would be less complicated and time consuming than training a more comprehensive treatment package that includes both behavioral and cognitive strategies, both of which may not be necessary for efficacy (e.g. in the treatment of depression; Jacobson *et al*. 1996). Given how little time may be available for training in low-resource settings, fewer strategies to train will allow for more time on each strategy and consequently less threat to treatment fidelity. Western therapies may have great value when applied in other cultures and resource-limited nations, but the field would benefit from additional implementation research to better understand the barriers and facilitators at all phases (Murray *et al*. 2013). Future work that explores low cost ways to conduct pre-pilot, pilot, and feasibility testing when delivering behavioral therapies in resource-limited settings is needed.
